# Minimally invasive spinopelvic “crab-shaped fixation” for unstable pelvic ring fractures: technical note and 16 case series

**DOI:** 10.1186/s13018-019-1093-1

**Published:** 2019-02-15

**Authors:** Akinori Okuda, Naoki Maegawa, Hiroaki Matsumori, Tomohiko Kura, Yasushi Mizutani, Hideki Shigematsu, Eiichiro Iwata, Masato Tanaka, Keisuke Masuda, Yusuke Yamamoto, Yusuke Tada, Yohei Kogeichi, Keisuke Takano, Hideki Asai, Yasuyuki Kawai, Yasuyuki Urisono, Kenji Kawamura, Hidetada Fukushima, Yasuhito Tanaka

**Affiliations:** 1 Department of Emergency and Critical Care Medicine, Nara Medical University, 840 Shijotyo, Kashihara City, Nara, 654-8522 Japan; 2 Department of Orthopedic Surgery, Nara Medical University, Kashihara, Nara, Japan; 3 Department of Orthopedic Surgery, Kashiba Asahigaoka Hospital, Kashiba, Nara, Japan; 4 Department of Orthopedic Surgery, Kura Hospital, Ikoma, Nara, Japan

**Keywords:** Crab-shaped fixation, Pelvic ring fracture, Sacral fracture, Minimally invasive surgery, Spino-pelvic fixation, Vertical displacement

## Abstract

**Background:**

Unstable sacral fractures are high-energy injuries and comprise polytrauma. Internal fixation to enable withstanding vertical loads is required to get up early from the bed after an unstable sacral fracture. We developed a new minimally invasive surgical (MIS) procedure for unstable pelvic ring fractures and reported it in Japanese in 2010. We presented our minimally invasive surgical technique of crab-shaped fixation for the treatment of unstable pelvic ring fractures and report on its short-term outcomes.

**Methods:**

Sixteen patients with unstable pelvic ring fractures (AO types C1, 2, and 3) were treated using crab-shaped fixation. All procedures were performed with the patient in the prone position through 5-cm skin incisions created bilaterally at the level of the posterior superior iliac spine. Four iliac screws were inserted and connected with two rods under the fascia. Percutaneous pedicle screws were inserted at L5 or L4 and connected to the iliac rod using offset connectors. Fracture reduction was then performed.

**Results:**

The average surgical time was 158 min (range, 117–230 min), with an intraoperative bleeding volume of 299 ml (range, 80–480 ml). Thirty-three pedicle screws and 64 iliac screws were implanted with no instance of malpositioning or perforation. A surgical site infection developed in 2 of the 16 cases. Both were deep methicillin-resistant *Staphylococcus aureus* infections, with the removal of the distal implants required in only one of these cases. Bony union was achieved in all patients, and all vertical displacements reduced by 7.0 mm, on average (range, 5.4–9.0 mm), to < 10 cm. Correction was retained in all cases.

**Conclusions:**

Crab-shaped fixation provides a feasible MIS approach for spinopelvic fixation, which allows good reduction of the vertical displacement of unstable pelvic ring fractures and bony union.

## Background

Spinopelvic fixation (SPF), using spinal instrumentation, is a method of rigid fixation for unstable pelvic ring fractures [1–3]. The use of lumbar pedicle and iliac screws (with a bridging rod) reduces the vertical length of SPF required, while providing a rigid fixation across the sacrum, sacroiliac joints, and ilium that can withstand the shear load of vertical forces during weight-bearing activities. Schildhauer et al. reported that rigid SPF led to good bony union, without loss of segmental correction [3]. Moreover, the recent use of a minimally invasive surgical (MIS) approach for SPF lowered the risk for infection and injury to the skin and soft tissues, while providing a higher rate of union than that with conventional open fixation [4, 5]. In 2014, we reported on our use, in Japan, of the “crab-shaped fixation” as a new MIS fixation technique for the treatment of unstable pelvic ring fractures, which reduces the vertical displacement of the fracture. We performed crab-shaped fixation on AO types C1 and C3 fractures with a large vertical dislocation or Denis zone III, AO–C2, and AO–C3 fractures [6]. For crab-shaped fixation, percutaneous pedicle screws were inserted bilaterally into the pedicles of L5 or L4, and four iliac screws were inserted bilaterally into the iliac crests, with titanium-alloy rods used to connect the screws both in the right–left and craniocaudal directions. In this way, the crab-shaped fixation can withstand not only vertical loading but also horizontal and rotational loading. The purpose of this study was to describe the surgical procedure for MIS crab-shaped fixation and to report on clinical outcomes.

## Methods

Patients consented to have their case data collected and used for publication.

The study group included 16 patients who were treated for an unstable pelvic ring fracture, between 2010 and 2016. All fractures were AO type C, including 1 type C1 fracture with > 10 mm of vertical displacement, 9 type C2 fractures, and 6 type C3 fractures (Table 1). The average age at the time of trauma was 50 years (range, 19–82 years). There were 8 men and 8 women, with an average Injury Severity Score of 27 (range, 14 to 41). The causes of trauma included the following: being struck by a vehicle (7 patients), including 1 case of being run over by a vehicle; motor vehicle accidents (6 patients); and a suicide attempt by jumping from a height (3 patients). Surgery was performed once patients were sufficiently stable.

**Table 1:** Clinical data of cases included in our study group

No.	Age	Sex	ISS	Cause of trauma	AO classification	Operation time (min)	Blood loss (g)	Vertical dislocation (mm)		Reduction rate (%)	Complication	Bony union
								Pre op	Post op			
1	21	F	17	Attempted suicide jump	C2	170	310	10.4	5	52		Union
2	29	F	27	Struck by a vehicle	C3	180	400	18.4	8.9	52		Union
3	19	F	14	Traffic accident	C3	168	480	4.8	4.8			Union
4	69	F	22	Struck by a vehicle	C3	131	200	5	5			Union
5	75	M	21	Struck by a vehicle	C2	172	200	2.4	2.4			Union
6	36	F	29	Attempted suicide jump	C2	157	320	1.5	1.5			Union
7	59	F	29	Traffic accident	C2	120	150	2	2		SSI (MRSA)	Union
8	64	M	26	Struck by a vehicle	C3	171	300	1.5	1.5			Union
9	50	M	29	Traffic accident	C2	117	250	12	6.5	46		Union
10	40	F	41	Run over by a vehicle	C3	184	450	2.2	2.2		Gluteal muscle necrosis, SSI (MRSA)	Union
11	46	M	22	Attempted suicide jump	C2	155	480	18	9	50		Union
12	82	M	38	Traffic accident	C2	120	80	10.5	5	52		Union
13	50	F	38	Traffic accident	C1	132	120	18.3	12	34		Union
14	59	M	24	Struck by a vehicle	C3	230	350	15	7.5	50		Union
15	51	M	26	Traffic accident	C2	150	267	15	7.5 (5.0)	50	Lt L5 nerve root pain remained.Re-operation performed.	Union
16	53	M	22	Struck by a vehicle	C2	175	430	1	1			Union

Case 15; *(5.0)*: vertical dislocation after secondary operation; *Lt*, left; *pre op*, preoperatively; *post op*, postoperatively; *M*, male; *F*, female; age, reported in years; *ISS*, injury severity score

**Fig. 1:**
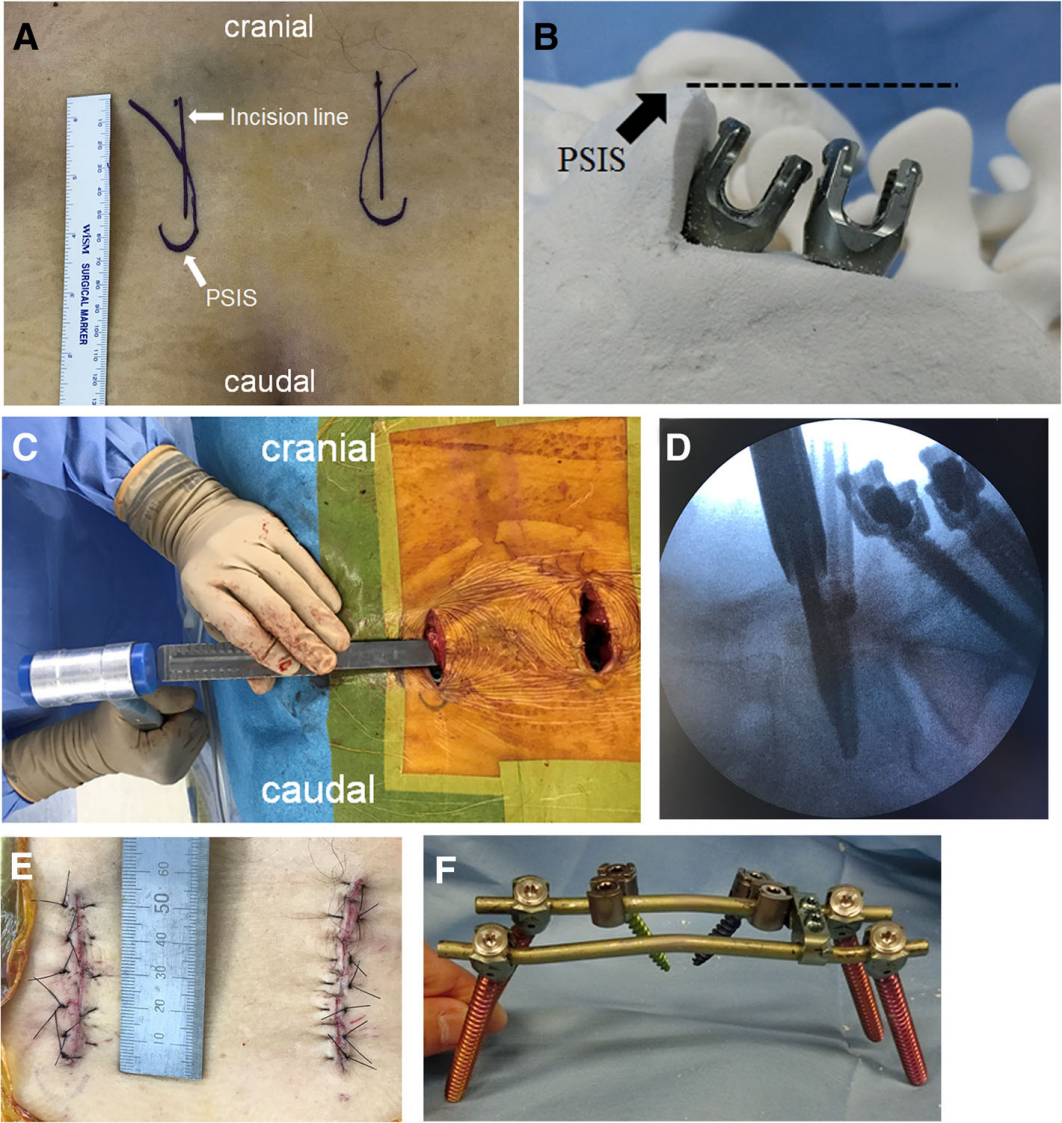
Surgical procedure using the crab-shaped fixation. **a** A 5-cm incision line is created along the posterior superior iliac spine (PSIS). **b** The PSIS is resected (2-cm depth and 3-cm length), leaving the top of the PSIS intact. The iliac screws are inserted from the resection site of the ilium, using a free-hand technique, with the screw heads set lower than the top of PSIS to prevent skin breakdown (dotted line). **c** The spinous process of S1 is cut under the fascia to allow a smooth insertion of the rod. **d** Percutaneous pedicle screws at L5 or L4 are inserted, via the same incision, under fluoroscopic guidance. **e** Postoperative wound closure. **f** The crab-shaped fixation is shown

All crab-shaped spinopelvic fixations were performed using an MIS approach. We made a 5-cm incision along the medial border of the posterior superior iliac spine (PSIS) (Fig. 1a). We then proceeded with resection of the iliac bone (3 cm in length, to a depth of 2 cm), preserving the superior surface of the PSIS (Fig. 1b). Bone resection was performed to ensure that there would be no protrusion of the heads of the screws, to prevent postoperative irritation and skin necrosis. We inserted two iliac screws on each side, using a free-hand technique with an equal distance between the screws. In each case, we used large iliac screws (> 7 mm in diameter and with a length ≥ 65 mm, as possible in each case). The iliac screws were set lower than the top of the PSIS, as shown by the dotted line in Fig. 1b. We also removed the spinous process of S1 using a chisel, below the fascia, in cases where the spine interfered with insertion of the rod connecting the left and right screws (Fig. 1c). We connected the two upper and lower rods using one or two transverse connectors. Percutaneous pedicle screws (PPSs) were then set into L5 (or L4), through the same incisions along the PSIS, under image guidance, using a C-arm fluoroscope (WHA-200 OPESCOPE ACTIVO, Shimadzu Medical; Fig. 1d). Offset connectors were installed in the connecting rods implanted in the iliac bone and were then connected to the PPSs. We then proceeded with vertical reduction of the fracture through distraction between the ipsilateral PPS and offset connector rod. It is difficult to confirm whether the reduction is appropriate in MIS as it is complicated to perform direct reduction. Therefore, we performed the reduction by being aware of the following three points: (1) confirming the reduction by palpating the step off and gap of the fracture site using a finger during reduction, (2) measuring the reduction distance in advance with preoperative CT and reducing by the same distance, and (3) confirming the reduction under fluoroscopy with the outlet view. When we reduce the transversal displacement, we perform compression between the right and left the iliac screws or offset connector and ipsilateral iliac screw as long as the reduction distance matches with that measured in advance with preoperative CT, and we confirm the reduction on AP view under fluoroscopy. With accumulated experience, we changed the pedicle screws that we use for the fixation to the PPS system for MIS. We first used the open pedicle screw system (USS2 poly-axial system, SYNTHES), subsequently changing to the percutaneous pedicle screw system (SpiRIT Pangea system, SYNTHES). Currently, we use the new percutaneous pedicle screw system (Matrix–X-tab system, Depuy-SYNTHES, or CREO-MIS, Globus).

The following variables were used to evaluate clinical outcomes: operative time, intraoperative bleeding volume, screw perforation and malpositioning, surgical site infection (SSI), rate of bony union, and rate of achieving vertical reduction of the fracture.

## Results

The average surgical time was 158 min (range, 117 to 230 min), with an intraoperative bleeding volume of 299 ml (range, 80 to 480 ml; Table 1). The incidence of malpositioning of the lumbar pedicle screws and perforation of the iliac screws was evaluated using computed tomography (CT) based on the classifications given by Rao et al. [7] and Koshimune et al. [5], respectively. Among the 16 cases, we implanted 33 pedicle screws and 64 iliac screws, with no incidence of screw malpositioning or perforation.

With regard to complications, SSI developed in 2 of the 16 cases. In both cases, the infections were deep methicillin-resistant *Staphylococcus aureus* (MRSA) infections. Treatment included debridement and negative pressure wound therapy (NPWT), with one patient requiring removal of the distal implants (left and right iliac screws), with the proximal implants remaining in situ (both iliac screws and L5 pedicle screws). In the other case, gluteal necrosis developed after transcatheter arterial embolization (TAE), which resulted in deep infection. The patient was treated with debridement and NPWT without the removal of the implants. After bony union was achieved at 3 months, all implants were removed and the infection was cured.

**Fig. 2:**
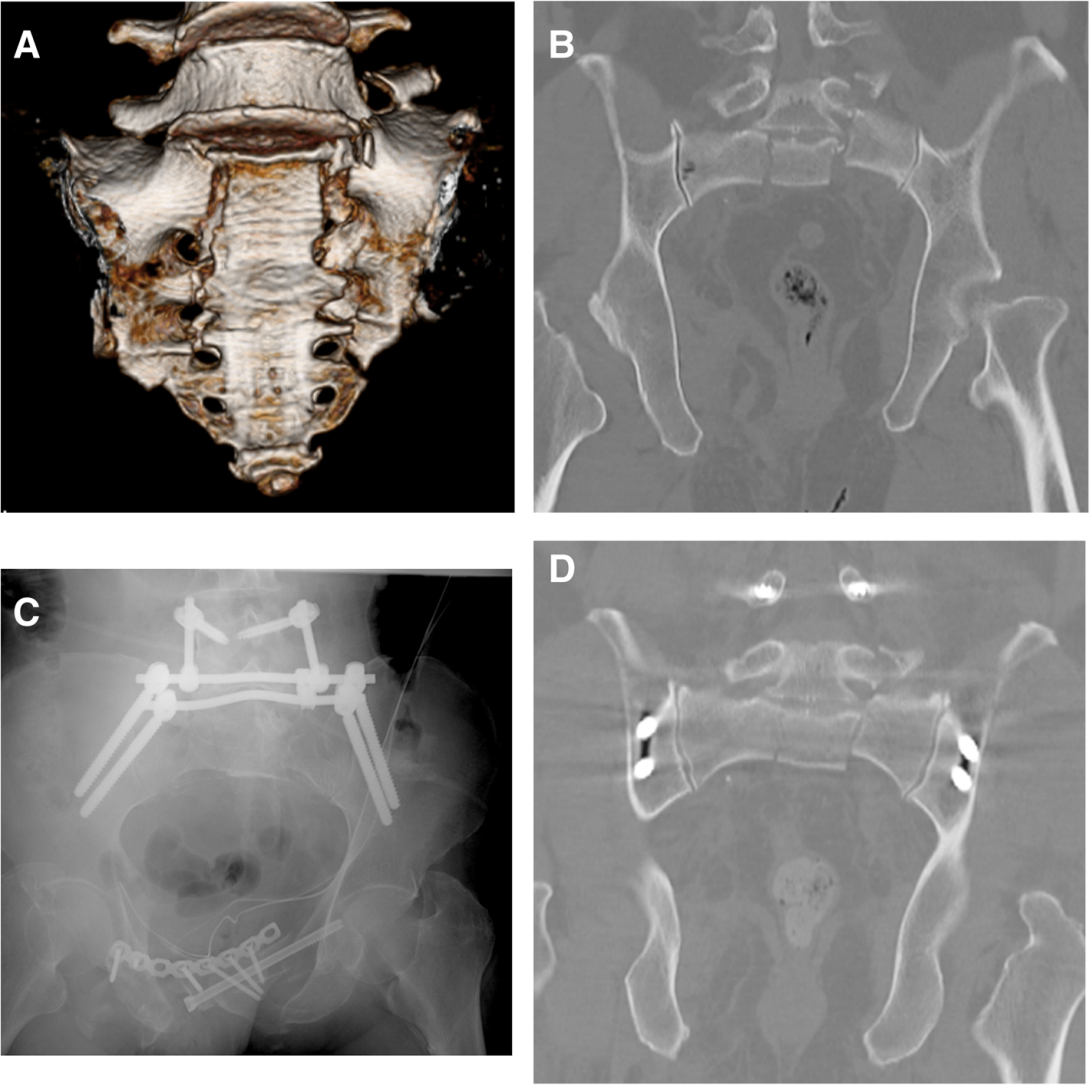
Case 15: a 45-year-old male who sustained an unstable AO type C2 pelvic ring fracture in a traffic accident, including a bilateral fracture of the Denis zone II of the sacrum and a 15-mm vertical dislocation (cranial direction). **a** Preoperative three-dimensional computed tomography (CT), showing the H-shaped sacral fracture. **b** The 15-mm vertical dislocation (cranial direction) is observable on the preoperative coronal plane CT. **c** Postoperative radiographs, showing the crab-shaped fixation. **d** Postoperative CT showing a residual 7.5-mm vertical dislocation (cranial direction)

**Fig. 3:**
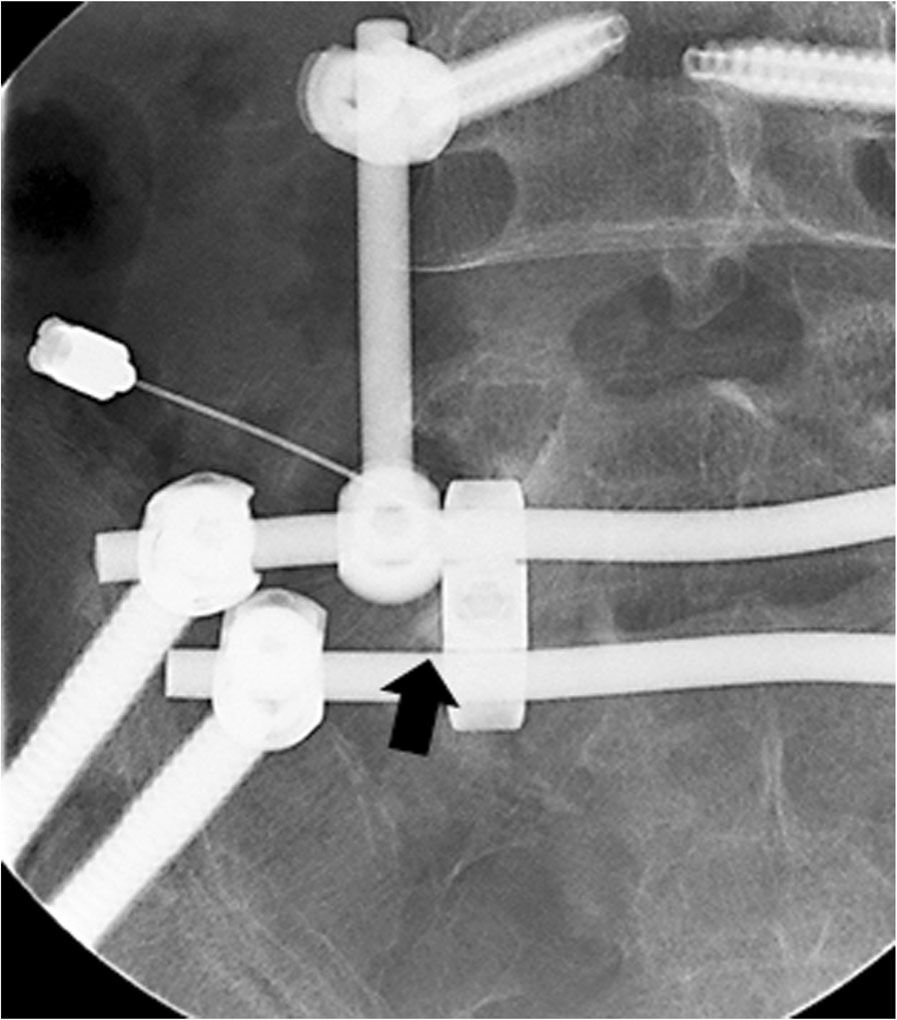
Radiculography of the left L5 nerve root, performed with a contrast agent, with root impingement in the lumbosacral tunnel (black arrow) observable on the posterior-anterior view

**Fig. 4:**
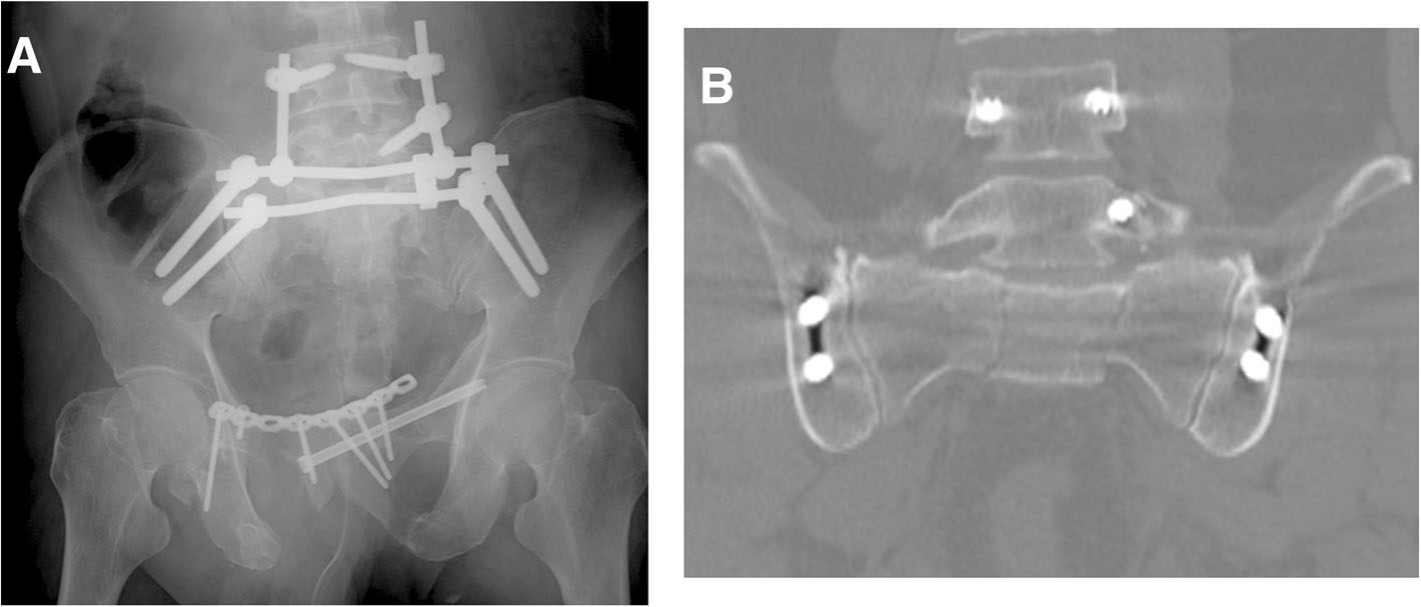
Radiograph and computed tomography (CT) images after surgery. **a** Anteroposterior radiograph, showing the left L5 pedicle screw, after secondary surgery, which is used to apply distraction to the fracture site (in combination with traction of the left lower limb). **b** Coronal CT after secondary surgery, showing the residual 5–7.5-mm residual vertical dislocation (cranial direction), but with resolution of the left L5 nerve root pain

Bony union was achieved in all 16 cases. Among the 16 cases, 8 presented with a vertical displacement ≥ 10 mm. The average reduction length was 7.0 mm (range, 5.4 to 9.0 mm), with an average reduction of the vertical displacement of 48% (range, 34% to 52%; Table 1). Among the 8 cases with vertical displacement, a residual displacement of ≥ 10 mm was identified in only one case, with no associated neurological symptoms from the lumbosacral plexus. In one of these cases (case 15), we reduced the vertical displacement from 15 mm to 7.5 mm, but the patient complained of pain caused by L5 nerve root entrapment (Fig. 2). Radiculography of the L5 nerve root, using a contrast agent, confirmed impingement of the nerve root, shown by the black arrow in Fig. 3, with stimulation of the nerve root reproducing the patient’s pain. As the residual vertical displacement of the fracture was compressing the nerve root, we proceeded with a repeat reduction of the fracture, successfully decreasing the residual displacement to 5 mm, with resolution of the neurological symptoms (Fig. 4). Of the eight patients who underwent fracture reduction, postoperative correction was maintained in all cases.

## Discussion

SPF and sacroiliac rod fixation (SIRF) have previously been described as MIS techniques with spinal instrumentation capable of reducing the vertical displacement of unstable pelvic ring fractures [4, 5]. Koshimune et al. performed SPF using the percutaneous spinal pedicle screw system [5], whereas Futamura et al. performed SIRF by linking each left and right iliac screw with a rod and connecting these to percutaneous S1 pedicle screws using offset connectors [4]. To perform the vertical reduction of the fracture, a Schanz screw was inserted into the L5 pedicle, and the vertical displacement of the fracture was reduced by distraction. However, the L5 pedicle screw was not used for fixation. Rather, the Schanz screw was removed after reduction while preserving the mobility of the L5/S1 facet joint [4]. The reported relevant outcomes for SPF and SIRF, respectively, were as follows: operative time, 208 min and 179 min; intraoperative blood loss, 290 g and 533 g; rate of SSI, 0% and 6.7%; and screw malpositioning, 0 and 1 screw. Bony union was achieved in all cases for both procedures. In contrast to conventional (open) sacral fixation, percutaneous SPF decreases operative time and intraoperative bleeding volume. It also has a low rate of postoperative infections while providing the rigid fixation needed for a high rate of bony union [5]. Bellabarba et al. reported that 42% of patients who underwent lumbosacral fixation using an open method required secondary surgery because of complications, which included deep infection (16%), implant irritation (11%), hematoma and seroma formation (10%), and skin ulceration (5%) [1]. Sagi et al. also reported that 16% of patients who undergo open lumbosacral fixation require implant removal due to an SSI [2].

In our case series, the use of the crab-shaped fixation further decreased operative time, in contrast to that for SPF and SIRF, with an intraoperative blood loss volume that was similar to that previously reported for SPF. Our rate of SSI (2 cases; 12.5%) was comparable to the rate for both SPF and SIRF. Regarding the nature of the SSI in our two cases, one was secondary to a gluteal necrosis that developed after TAE of the superior gluteal artery at the surgical site. The other SSI case was an MRSA carrier.

The crab-shaped fixation provided sufficient rigidity to achieve bony union in all cases, with no loss of correction, even in the two cases of SSI without complete removal of implants by using NPWT. Therefore, the crab-shaped fixation provides a feasible MIS alternative for spinopelvic fixation.

Lumbosacral plexus symptoms can develop when a strong traction force is applied to the sacrum for reduction of the vertical displacement of the fracture. The L5 nerve root may also be compressed in the lumbosacral tunnel with AO type C, which includes a vertical displacement of the Denis zone I or II of the sacrum of ≥ 10 mm [6, 8]. Therefore, fracture reduction should aim to decrease the vertical displacement to < 10 mm. Among our 16 cases, 1 patient developed L5 nerve root pain secondary to compression of the root in the lumbosacral tunnel, despite a reduction in the vertical displacement of the AO type C2 (sacral H-shaped fracture) from 15 mm preoperatively to 7.5 mm postoperatively (Table 1, case 15). We performed secondary surgery to further reduce the vertical displacement from 7.5 mm to 5.0 mm, which was achieved by using lower limb traction in combination with distraction using instrumentation. Based on this experience, it might be necessary to reduce the sacral vertical dislocation to within 5 mm.

The interpretation of our findings should be considered within the limitations of our study, which included a small number of cases; the absence of biomechanical data and the need for implant removal after bony union as it provided fixation across the L5/S1 facet joint. We may further evaluate the biomechanical data of crab-shaped fixation focusing on the appropriate or minimum size and length of the iliac screw to endure shear load force.

## Conclusion

Crab-shaped fixation provides a feasible MIS approach for spinopelvic fixation that provides sufficient rigidity to achieve bony union in the treatment of unstable pelvic ring fractures. This technique provides an easy way to achieve good reduction of the vertical displacement of the fracture.

## Abbreviations

CT: Computed tomography
MIS: Minimally invasive surgery
MRSA: Methicillin-resistant *Staphylococcus aureus*
NPWT: Negative pressure wound therapy
PPS: Percutaneous pedicle screw
PSIS: Posterior superior iliac spine
SIRF: Sacroiliac rod fixation
SPF: Spinopelvic fixation
SSI: Surgical site infection
TAE: Transcatheter arterial embolization
